# Astrocytic Mechanisms Explaining Neural-Activity-Induced Shrinkage of Extraneuronal Space

**DOI:** 10.1371/journal.pcbi.1000272

**Published:** 2009-01-23

**Authors:** Ivar Østby, Leiv Øyehaug, Gaute T. Einevoll, Erlend A. Nagelhus, Erik Plahte, Thomas Zeuthen, Catherine M. Lloyd, Ole P. Ottersen, Stig W. Omholt

**Affiliations:** 1 Centre for Integrative Genetics (CIGENE), Norwegian University of Life Sciences, Ås, Norway; 2 Department of Mathematical Sciences and Technology, Norwegian University of Life Sciences, Ås, Norway; 3 Centre for Molecular Biology and Neuroscience, Institute of Basic Medical Sciences, University of Oslo, Blindern, Oslo, Norway; 4 Nordic Centre for Water Imbalance Related Disorders, University of Oslo, Blindern, Oslo, Norway; 5 Institute of Cellular and Molecular Medicine, The Panum Institute, University of Copenhagen, Copenhagen, Denmark; 6 Auckland Bioengineering Institute, The University of Auckland, Auckland, New Zealand; 7 Department of Animal and Aquacultural Sciences, Norwegian University of Life Sciences, Ås, Norway; University College London, United Kingdom

## Abstract

Neuronal stimulation causes ∼30% shrinkage of the extracellular space (ECS) between neurons and surrounding astrocytes in grey and white matter under experimental conditions. Despite its possible implications for a proper understanding of basic aspects of potassium clearance and astrocyte function, the phenomenon remains unexplained. Here we present a dynamic model that accounts for current experimental data related to the shrinkage phenomenon in wild-type as well as in gene knockout individuals. We find that neuronal release of potassium and uptake of sodium during stimulation, astrocyte uptake of potassium, sodium, and chloride in passive channels, action of the Na/K/ATPase pump, and osmotically driven transport of water through the astrocyte membrane together seem sufficient for generating ECS shrinkage as such. However, when taking into account ECS and astrocyte ion concentrations observed in connection with neuronal stimulation, the actions of the Na^+^/K^+^/Cl^−^ (NKCC1) and the Na^+^/HCO_3_^−^ (NBC) cotransporters appear to be critical determinants for achieving observed quantitative levels of ECS shrinkage. Considering the current state of knowledge, the model framework appears sufficiently detailed and constrained to guide future key experiments and pave the way for more comprehensive astroglia–neuron interaction models for normal as well as pathophysiological situations.

## Author Summary

A key experimental observation associated with the astroglia–neuron interaction is the shrinkage of the extracellular space (ECS) that occurs in response to enhanced neuronal activation. Although well documented to be present in mammalian brains, this phenomenon has resisted a proper explanation since it was first reported. We present here a mathematical conceptualization that may explain the main mechanisms behind ECS shrinkage and provide a framework for a theoretical-experimental research programme that may help to reach a consensus explanation. Effective clearance of K^+^ is essential for normal brain function because an inappropriate increase in extracellular K^+^ will enhance neuronal excitability and promote neuronal afterdischarges and increase the probability of epileptic episodes. The shrinkage of the ECS usually appears in conjunction with K^+^ clearance and must be taken into account in a model of how astrocytes clear excess K^+^ following trains of action potentials. The present model allows us to make several clear and testable predictions addressing the relationship among potassium clearance, water transport, and ECS shrinkage. Among these are predictions concerning water transport functions of aquaporins in astrocytes, involvement of cotransporters in potassium clearance, and effects of particular knockouts on ECS shrinkage and ion concentrations.

## Introduction

The term *astrocyte* was introduced by Lenhossék (1893) to describe the starshaped neuroglial cells first discovered by Otto Deiters in the mid 19^th^ century (reviewed by Cajal [1]). These cells constitute the most abundant type of *macroglia* in the brain [2]. During most of the 20^th^ century they were considered passive bystanders of neural activity. The picture that is now materializing is that astrocytes are critically involved in modulation of excitatory and inhibitory synapses by removal, metabolism, and release of neurotransmitters [3], homeostatic maintenance of extracellular K^+^, H^+^ and glutamate levels [4], supply of energy substrates for the neurons [5], neuronal pathfinding during development and regeneration [6], and trophic modulation of neural repair and axon regrowth following injury. In addition, astrocytic cells themselves seem to have key roles in central nervous system disorders from neuropathic pain and epilepsy to neurodegenerative diseases such as Alzheimer's as well as schizophrenia, depression, and other psychiatric disorders [7]. Astrocytes are known to interact extensively with neuronal brain elements as well as the vasculature to form functional compartments controlling communication pathways, thresholds and plasticity. This allows the formation of astrocyte networks which serve to match neural activity and blood flow, regulating neuronal firing by coordinated astrocytic Ca^2+^ signaling [8]. By these means, astrocytes establish an extensive functional architecture of the brain with roles that remain to be fully explored.

A good proximal understanding of the normal behavioral repertoire of astrocytes is a prerequisite for understanding the large number of pathophysiological conditions that may arise from dysfunctional situations as well as for assessing the potential of the astrocyte as a therapeutic target. However, our current concepts of even long known aspects of this repertoire are quite obscure. Perhaps the most prominent illustration of this is our lack of understanding of the mechanisms that underlie the substantial shrinkage (∼30%) of the local extracellular space that is observed 10–20 seconds after neuronal activation under experimental conditions [9]–[16].

The ECS shrinkage phenomenon is closely linked to potassium clearance as well as water transport processes. Potassium ions are discharged from excited neurons with each action potential and each excitatory postsynaptic potential [17]. During low frequency firing, the most common mode of operation of neurons, the ATP-fueled membrane 3Na/2K pump located in the neuronal membrane seems able to keep up with the outflow of potassium so that the ions are returned to the neuron [17]. However, during high frequency firing the major sink of excess ECS K^+^ is the surrounding astrocytic tissue [13],[17]. Several potassium clearance mechanisms have been identified [12], [14], [18]–[22], but their relative importance in various contexts has been under intense debate for several decades [9], [10], [19]–[21],[23].

Concerning water transport, the influx of potassium (and accompanying anions ensuring space-charge neutrality) [24] causes the set-up of an osmolarity gradient which drives water from the ECS into the astrocyte [12],[25]. The water transport through the astrocyte membrane is thought to be mainly taken care of by perisynaptic aquaporin (AQP4) channels [26],[27]. However, this is far from settled. We do not have conclusive data on the rate of water transport through AQP4 or other water channels during low-frequency firing. Assuming that perisynaptic AQP4 is involved in water transport during high-frequency firing, we are not yet in position to predict in which direction the water flows through AQP4 channels [28],[29].

The above considerations suggest that the ECS shrinkage phenomenon deserves serious attention because a quantitative understanding of the underlying regulatory anatomy is likely to reveal new and important insights concerning the concerted actions of astrocyte membrane processes as well as the astroglia-neuron interaction. Active transport of sodium and potassium by the Na/K/ATPase pump, ion transport (potassium, sodium and chloride) between the astrocyte and the ECS compartment through passive ion channels and water transport through aquaporin (AQP4) channels in the perisynaptic membrane areas [29] are all well documented membrane processes that are likely to be major contributors to ECS shrinkage. Thus, a key question is how much of the observed shrinkage can be accounted for by the concerted action of these three processes. If the answer is that contributions from one or more additional transporters are needed, the next questions are which these are and what their contributions in quantitative terms are. There is no apparent consensus concerning the relative contributions to potassium clearance and volume regulation from various types of transporters, whose cardinal feature is the ability to move solutes against an electrochemical gradient [30]. Several inorganic osmolyte transporters are known to contribute to cell volume regulation. Of potential importance are the sodium-potassium-chloride cotransporter (NKCC1), the potassium-chloride cotransporter (KCC1), the chloride/bicarbonate anion exchangers (AE), the sodium-bicarbonate cotransporter (NBC), the sodium-driven chloride/bicarbonate exchanger (NDCBE) and the sodium/hydrogen exchangers (NHE) (reviewed by Kettenmann & Ransom [30]).

The above state of affairs motivated us to develop a mathematical conceptualization of the ECS shrinkage phenomenon during normal neuronal activity to see which hypotheses concerning potassium clearance and membrane water transport mechanisms can be reconciled with available data and to identify crucial new experiments that can bring us forward. As long as we model the relative ECS shrinkage by astrocyte membrane processes we are not dependent on a precise geometrical specification of the region of interest [19]. It suffices to specify the ratio between ECS volume and the associated astrocyte volume and the ratio between astrocyte membrane area and astrocyte volume. Average experimental values for these two ratios in the mammalian brain are available [31],[32], and they allow model predictions to be valid for tissue volumes large enough to reflect available measurements of the relative shrinkage as well as ion concentrations.

To be able to use temporal information to estimate parameters, validate models and generate predictions, we developed within a common framework five dynamic models to describe and analyze ECS shrinkage as a function of neural activity and astrocyte membrane processes identified above. It should be emphasized that we do not model the processes underlying neuronal activity explicitly, but mimic the effects of neural activation as perturbations of an ECS steady state situation. More precisely, we assume that a temporary increase in neuronal activity (increased frequency of action potentials) results in a transient moderate increase in K^+^ efflux from the neuron to the ECS [9], [10], [12], [14]–[16],[33],[34], and that the concomitant influx of Na^+^ and Cl^−^ ions from the ECS into the neuron [24],[35] is dominated by Na^+^ to such a degree that the Na^+^ influx and K^+^ efflux can be considered to be equal. Even though this premise may be more valid for white matter than for grey matter regions, we have found it worthwhile to assume that it also reflects the grey matter situation quite well.

**Figure 1:**
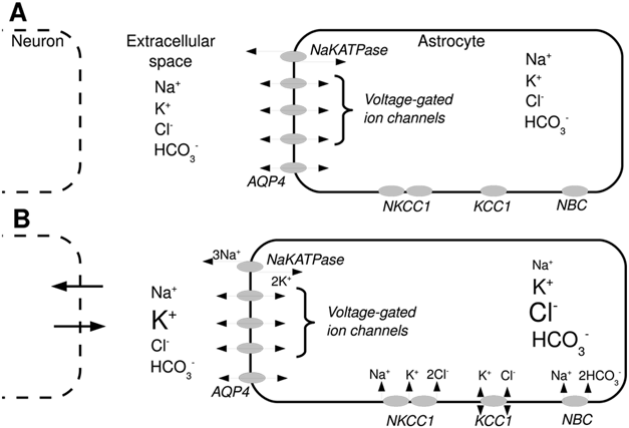
Outline of basic model premises in baseline and excited states. (A) In the baseline condition the neuron is assumed to be silent, i.e. there is no net exchange of ions across the neuronal membrane, cotransporters KCC1 and NBC are approximately in equilibrium and NKCC1 is assumed to be non-operative. (B) The neuron is treated as a black box, but in the excited state the neuron's contribution to the ECS shrinkage phenomenon is considered to be through its potassium efflux and a sodium influx (arrows) during high-frequency firing and through the reversal of these fluxes after the high rate of firing has abated. KCC1, NBC and NKCC1 are assumed to be operative in the excited state (arrows indicate the direction of ion flux, note that KCC1 may transfer ions both ways). Changes in font size and astrocyte size refer to the magnitude of changes from baseline to excited state.

The five model configurations are used to test several different wild type as well as gene knockout or knockdown situations (see Figure 1, Methods, and Text S1 for description of the model and its premises). They are based on five different hypotheses concerning which set of concerted astrocyte membrane processes can account for the ECS shrinkage phenomenon: H1: Na/K/ATPase pump+passive ion transport (potassium, sodium and chloride) between the astrocyte and the ECS compartment+osmotically driven water transport through the astrocyte membrane; H2: the cotransporter KCC1+H1; H3: the cotransporter NBC+H1; H4: the cotransporter NKCC1+H1; and finally (based on the outcomes from testing H1 to H4) H5: NKCC1+NBC+H1.

We did the testing through five model configurations mc1–mc5 corresponding to the hypotheses H1–H5. To account for measurement inaccuracies and uncertain parameter value estimates we solved each model configuration 50 000 times by sampling parameter values and a subset of initial baseline ion concentrations uniformly from specified regions, while the remaining baseline concentrations as well as some of the parameter values were calculated from this subset by use of the model's steady state equations. Details about this procedure and the accompanying indirect parameterization are given in Methods and Text S1.

## Results

### Tests for Empirical Consistency

**Figure 2:**
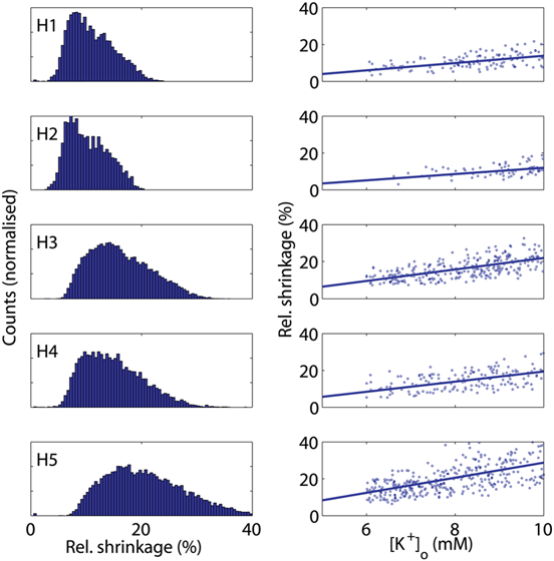
Distribution of volume shrinkage and the potassium-volume shrinkage relation for different models. Left panels: Normalised histograms of the distributions of relative ECS volume shrinkage (in %) for the five model configurations (mc1–mc5). The results were obtained by repeated numerical solution of steady state equations and only those parameter sets that satisfied the imposed ion concentrations constraints were used (see Methods). Right panels: Corresponding scatter plots of excited state relative shrinkage and potassium concentrations using the same data as in the left panels. Since the upper limit of the shown relative shrinkage and the lower limit of the shown [K^+^]_o_ are set to 40% and 5 mM, respectively, some of the data are not displayed. Best linear fits are shown, and the corresponding slopes are 1.97, 1.70, 3.11, 2.74 and 4.08, respectively. Moreover, for reasons of visualization only 2% of the data are depicted.

In order to constrain the model behavior by observational data, we kept only parameter sets giving baseline and excited state ion concentrations in accordance with a list of concentration range constraints designed from the available literature (specified in Methods). For each of the five model configurations we calculated the percentage of such empirically consistent parameter sets relative to the total number of simulations (the consistency rate), the relative volume shrinkage and the extracellular potassium concentrations (Table 1). The frequency distribution of the predicted ECS shrinkage for each model configuration is depicted in the left panel of Figure 2, and corresponding regression plots showing the relation between relative ECS shrinkage (in baseline+excited states, only data points corresponding to excited state shown) and ECS potassium concentration ([K^+^]_o_) are shown in the right panel. The slopes of the regression curves, listed in the legend to Figure 2, express the amount of ECS shrinkage obtained for a given [K^+^]_o_ for each model configuration. In order to retain the empirically most relevant parameter sets, we then selected the sets capable of also generating ECS shrinkage in the range 25–35%, and repeated the calculations of consistency rate, the relative volume shrinkage and [K^+^]_o_ (Table 1).

**Table 1:** Consistency rates and relative volume shrinkage.

	EC1		EC2	
	CR_1_ (%)	RS_1_ (%)	CR_2_ (%)	RS_2_ (%)
H1	11.9	10.8±4.0	0	
H2	6.6	10.1±3.6	0	
H3	30.1	15.9±5.4	2.0	27.2±1.8
H4	16.1	14.6±5.4	0.7	27.4±2.1
H5	34.6	20.5±7.3	7.7	28.9±2.7

Consistency rates (CR) and excited state relative volume shrinkage (RS) given by model configurations mc1 to mc5 under the two different constraints situations (ion concentrations only (EC1) and ion concentrations plus ECS shrinkage in the 25–35% range (EC2)).

We observe a dramatic reduction in the number of parameter sets in the second group compared to the first. The results suggest that neither the three basic membrane processes (Na/K/ATPase pump, passive ion transport, osmotically driven water transport) (Hypothesis 1) nor the added contribution from KCC1 (Hypothesis 2) are likely able to generate ECS shrinkage in the empirically observed range [9], [10], [12]–[16] (Table 1). In contrast, 2% and 0.7% of the 50000 parameter sets passed both the ion concentration and the ECS shrinkage test for the two model configurations reflecting Hypotheses 3 and 4, respectively (Table 1). There is only a marginal difference between the relative volume shrinkage predicted by model configurations 3, 4, and 5 (Table 1). However, the combined action of NBC and NKCC1 (mc5) gives a four- to tenfold increase in the percentage of empirically consistent parameter sets compared to when these cotransporters are operating alone. The fact that the ECS shrinkage phenomenon is observed across a whole range of experimental contexts [9],[12],[13],[15],[16] indicates that it is generated by a wide set of parameter configurations.

The model underlying H4 (mc4, only NKCC1) predicts a small decrease in [Cl^−^]_o_ during stimulation (compared to [9],[13] whereas mc3 and mc5 associated with H3 and H5 (NBC only and NBC+NKCC1, respectively) predict an increase in [Cl^−^]_o_ (Figure S4), which is in accordance with empirical observations [9],[13].

The above results were based on the assumption that there is a 1-to-1 relationship between the magnitudes of potassium neuronal efflux and sodium influx at high neural activity (see Text S1 for a simple calculation in support of this). However, relaxation of this assumption, either by reducing the potassium or increasing the sodium flux, and by compensating the change by adding an inwardly directed chloride flux to ensure neuronal and extracellular electroneutrality, did not cause dramatic changes (Figure S2, Figure S3, and Text S1).

In order to establish a common parameter set for the baseline state for all five cases H1–H5, we assumed the permeabilities of cotransporters NKCC1 and KCC1 were zero in the steady state and upregulated as functions of time along with increasing neural activity. Assuming constitutive action of these cotranporters revealed no significant change in the frequency distribution of predicted ECS shrinkage in the H2 case (KCC1 only) and a slight change towards the left (i.e. smaller predicted shrinkage) in the frequency distribution in the H3 and H5 cases (NKCC1 only and NKCC1+NBC, respectively, see Figure S1 and Text S1).

### Robustness Issues

**Figure 3:**
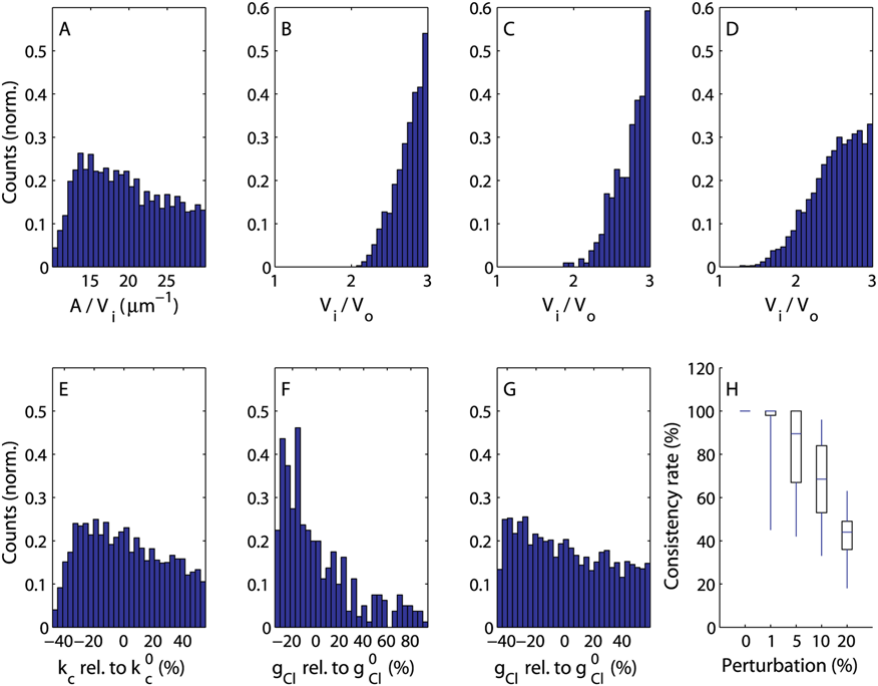
Parameter sensitivities and robustness to simultaneous parameter perturbation. (A–G) Normalised histograms of parameter values that satisfy both the imposed ion concentration constraints and an ECS shrinkage in the range of 25–35% for mc3, mc4 and mc5. (A) *A/V_i_* (astrocyte area to volume ratio) for mc5 (mc3 and mc4 display roughly the same pattern), (B–D) *V*_i_/*V*_o_, (astrocyte volume to ECS volume ratio) for models mc3, mc4 and mc5, respectively, (E) *k*_C_ (magnitude of neuronal potassium efflux/sodium influx) for mc5 (mc3 and mc4 display roughly the same pattern), (F,G) *g*_Cl_ (chloride conductance) for models mc4 and mc5, respectively (mc3 displays a uniform pattern). (H) For each of 100 parameter sets randomly selected from the 5000 sets associated with H5 we sampled randomly 100 new parameter sets where all parameter values were within a given percentage range of the original value, and for each of these 10000 sets we estimated the remaining parameters by use of the steady state equations as described. The figure shows the percentage of the empirically consistent parameter sets (satisfying prequalification set 2) for mc5 that still satisfy all imposed constraints after having been perturbed by uniformly resampling of each directly estimated parameter value from the specified percentage range around the initial parameter value (percentages corresponding to mc3 and mc4 are similar). The horizontal lines within the boxes indicate median, boxes comprise data that lie within quartiles and full vertical lines (“whiskers”) indicate the spread of the data (all data are included).

Focusing on the parameter sets meeting both the ion concentration and the ECS shrinkage constraints, we then asked whether there were marked differences between the three hypotheses H3, H4 and H5 in terms of distributions of those parameter values that were deliberately set in each simulation. The distribution of the *A/V_i_* values (the ratio of astrocyte membrane area to astrocyte volume) appears to be quite uniform around the estimate 18.9–33 µm^−1^ provided by Hama and Arii [32] based on computer electron tomography and stereo-photogrammetry, which suggests that this parameter can vary quite substantially without giving empirically inconsistent results (Figure 3A). However, all three distributions of the astrocyte volume to ECS volume ratio are skewed to the right of the sampling domain (Figure 3B–D). It appears difficult to obtain empirically consistent predictions when this ratio is below 2.0 for H3 and H4. The distribution of the ratio following from H5 allows somewhat more flexibility, and is in nice accordance with the reported ratio estimate of 2.3 [31].

The unimodal distribution of the magnitude of neuronal potassium efflux/sodium influx (*k*_C_) associated with H5 suggests that this parameter can also vary substantially within the constrained set (Figure 3E) (H3 and H4 give roughly the same pattern). The distribution of the chloride conductance (*g*_Cl_) in H4 is somewhat skewed to the left (Figure 3F), while H3 and H5 are associated with almost uniform distributions (Figure 3G). However, the skewness is too marginal to draw biological conclusions from. The three distributions for sodium conductance (*g*_Na_), the two distributions for NBC conductance (*g*_NBC_) in mc3 and mc5, the two distributions of the NKCC1 flux parameter (*g*_NKCC1_) in mc4 and mc5 and the ion concentration distributions are all very uniform (not shown).

We further tested whether the qualified parameter sets associated with the three hypotheses were robust to simultaneous moderate perturbations of the parameters in terms of empirical consistency. The overall impression is that all model configurations are structurally stable with regard to empirical consistency over a wide range of parameter constellations (Figure 3H).

### Study of the NKCC1^−/−^ Situation

**Figure 4:**
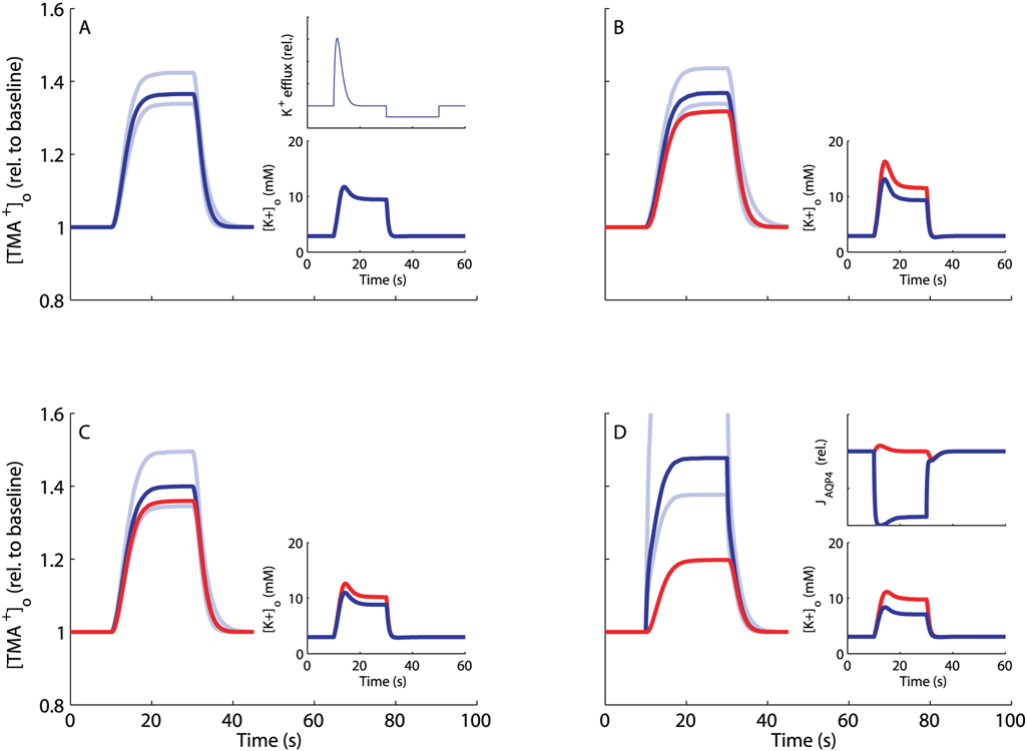
Predicted dynamics of [TMA^+^]_o_ and [K^+^]_o_ in wild types and NKCC1 knockouts. Predicted activity-dependent ECS volume dynamics in wild types (blue) and NKCC1 knockouts (or bumetanide-treated) (red) obtained by numerical solution of model equations (3) (see Methods) from *t* = 0 s to *t* = 100 s with enhanced neural activity for 10 s<*t*<30 s, using the parameter sets that satisfy all imposed constraints for (A) mc3, (B) mc4, (C) mc5. (D) Wild type and NKCC1 knockout of mc5 with active water transport through NKCC1 (in addition to AQP4-mediated passive water transport). In all plots, curves drawn with strong contrast are median values and the upper and lower curves drawn with weaker contrast define the boundaries between which 80% of all values in the used parameter sets fall. Lower insets show corresponding temporal evolution of the median ECS potassium level. (A) The upper inset displays the time-dependent potassium efflux (resp. sodium influx) rate profile (beta distribution with *a* = 2 and *b* = 16.0304) that is optimized for each model to yield potassium profiles in accordance with empirical observations (see Methods). The profiles corresponding to (B–D) resemble the one in (A) very closely (values for *b* are 15.18, 14.56 and 14.59, respectively). (D) The upper inset shows the AQP4-mediated water flux relative to zero. In mc3, NKCC1 is not included, hence NKCC1 knockout yields identical results as the wild type (A).

We mimicked a bumetanide-induced NKCC1^−/−^ situation [20],[34],[36] in models mc4 and mc5. The results are displayed in Figure 4 as predicted changes in the concentration of tetramethylammonium (TMA^+^) as a function of time, TMA^+^ being the state of the art assay for measuring ECS shrinkage. For both model configurations the reduction in mean relative shrinkage is predicted to be very moderate (Figure 4B and 4C). Assuming the wild type to have a relative ECS shrinkage of 30%, the results suggest that bumetanide-treated acute brain slices [37] will show a relative shrinkage of 26–27%, but with maximal [K^+^]_o_ values above those of the wild type (Figure 4, insets). The predicted increase in [K^+^]_o_ in knockout individuals relative to the wild type is much larger for H4 than H5. This result is in qualitative agreement with several experimental studies. Mice NKCC1^−/−^ astrocyte cell culture studies [21] and studies on brains, brain slices or dissected nerves [15],[34],[38] exhibit substantially reduced or close to zero shrinkage following bumetanide treatment. However, we predict a less pronounced shrinkage effect.

### Water Flow Direction through AQP4 and Water Transport by NKCC1

The direction of the water flow through the AQP4 and other passive channels as a function of neural activity has been given considerable attention [28],[29], but the issue still seems unresolved. The wild type models reflecting H3, H4 and H5 take as a premise that increased neural activity will cause a temporary net flow of water from the ECS into the astrocyte through the passive channels during ECS shrinkage, and subsequent release of this water back to the ECS as the system returns to steady state. However, this scenario depends on the assumption that no water is being transported actively with the ions through the NKCC1 cotransporter – an assumption that is currently under much debate [39]–[41]. If there is such a water transport through NKCC1 during the potassium clearance phase, this import will cause the swelling of the astrocyte, and the subsequent shrinkage of the astrocyte will be mainly caused by water flowing back into the ECS through the AQP4 channels. This motivated us to test the consequences for ECS shrinkage of knocking out a water-carrying NKCC1 cotransporter (by allowing NKCC1 active water cotransport in the wild type model associated with H5 (mc5), see Methods). The predicted reduction in relative shrinkage becomes dramatically more pronounced in this case (Figure 4D). The difference is so large that a well designed bumetanide experiment on acute slices showing a small, but statistically significant effect of inhibiting NKCC1 would strengthen the claim that NKCC1 in the astrocyte membrane does not transport water in connection with potassium clearance.

### The AQP4^−/−^ Situation

**Figure 5:**
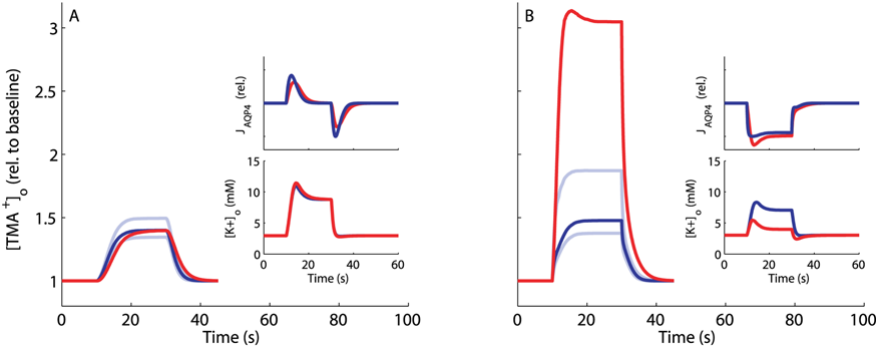
Predicted dynamics of [TMA^+^]_o_ and [K^+^]_o_ in wild type and AQP4 knockout. Predicted activity-dependent ECS volume dynamics in wild type (blue) and AQP4 knockout (red) obtained by numerical solution of model equations (3) (see Methods) from *t* = 0 s to *t* = 100 s with enhanced neural activity for 10 s<*t*<30 s, using the parameter sets that satisfy all imposed empirical constraints for mc5 without (A) and with (B) active water transport through NKCC1. For figure details, see legend to Figure 4. In both plots, the upper insets show the AQP4-mediated water flux relative to zero, and lower insets display the dynamics of ECS potassium levels.

Total water permeability *L_p_* of the astrocyte membrane includes the permeability of the aquaporins AQP4 as well as a contribution from other passive water channels. To account for the continued contribution of passive channels, we mimicked the AQP4^−/−^ situation by reducing the total permeability by 80% [42] in mc5. This led to negligible changes in ECS relative shrinkages compared to the wild type (Figure 5A). However, if we allow NKCC1 to transport water (see previous section), the revised model predicts a dramatic increase in relative shrinkage in an AQP4^−/−^ situation (Figure 5B). Thus, this prediction suggests another indirect test of whether NKCC1 in the astrocyte membrane does or does not transport water in connection with potassium clearance.

## Discussion

According to our results, the shrinkage phenomenon is a consequence of the concerted action of several processes. If we require that our models produce ion concentrations in the excited state that are consistent with empirically observed levels within the narrow time window relevant for the experimental ECS shrinkage observations, the presence of either NBC or NKCC1 or both seems necessary in order to produce ECS shrinkage to the extent reported in the literature. However, even without NBC and NKCC1 a potassium pulse followed by a removal of sodium from the ECS by the neuron, astrocyte uptake of chloride and other ions through passive channels [15],[25],[34], the action of the Na/K/ATPase pump, and the presence of AQP4 channels seem sufficient for generating ECS shrinkage as such. In fact, the associated model configuration (mc1) is capable of generating ECS shrinkage in the 25–35% range, but only with concomitant [K^+^]_o_ levels well above those actually observed under normal physiological conditions (see below).

The main reason why cotransporters may enhance the shrinkage phenomenon is that they cause a transient increase of intracellular osmolarity through transfer of ions to the intracellular compartment. In the NBC case, the increase in [K^+^]_o_ implies a depolarization of the astrocyte membrane which is sensed by the cotransporter to yield increased influx of both Na^+^ and HCO_3_^−^ [43]–[45]. The NKCC1 cotransporter responds to relative changes in ion concentrations (see Methods), and as [Na^+^]_o_ and [Cl^−^]_o_ only change marginally during neural activity (changes are ∼5%), the empirically observed 70–200% increase in [K^+^]_o_ [9], [11]–[13],[15] appears to be the main factor causing increase in rate of ion transfer through NKCC1. Despite the formal similarity between NKCC1 and KCC1, the results obtained from mc2 indicate that KCC1 does not contribute substantially to ECS shrinkage. This is because mc2 predicts a net outward flux of K^+^ and Cl^−^ through KCC1 under empirically relevant ECS and astrocyte ion concentrations (data not shown). The result is in accordance with the common conception that NKCC1 and KCC1 have opposing roles concerning maintenance and regulation of cell volume (see Gagnon [46] for references).

Our results suggest that among H3, H4 and H5, the last one appears as the most probable one. The operating modes of NKCC1 and NBC are quite different, they are triggered by changes in [K^+^]_o_ and by [K^+^]_o_–induced membrane depolarization, respectively, and sodium is the only ion species that is transported by both. Thus, due to this difference in operation, NBC and NKCC1 can be said to be complementary. Their combined action together with the three basic membrane processes give a much higher number of parameter sets with empirically relevant predictions than the other two hypotheses. Thus H5 provides a more realistic distribution of the astrocyte volume to ECS volume ratio than the other two, and in contrast to H4 it predicts the observed increase in [Cl^−^]_o_ during shrinkage. It should also be noted that H5 predicts a much stronger relation between degree of the ECS shrinkage and [K^+^]_o_ than the other two (Table 1), which implies that a given ECS shrinkage is obtained with a smaller number of discharged potassium ions from excited neurons. This may be of functional significance.

The moderate effect of knocking out NKCC1 suggests that a given actor may be an important causal agent for a specific phenomenon and yet leave only subtle signatures when removed. Then why is the predicted shrinkage reduction after NKCC1 knockout so slight? In the mc4 case, the removal of NKCC1 causes a higher [K^+^]_o_ in the excited state (Figure 4B and 4C, insets). As in the mc1 case with no constraint on [K^+^]_o_ , this will cause a relatively large shrinkage. In the mc5 case, the NKCC1^−/−^ induced increase in [K^+^]_o_ is lower. However, it is high enough to cause a depolarization of the astrocyte membrane and thus an increased NBC-mediated ion flux buffering the impact from knocking out NKCC1. In this sense NBC can be said to partly compensate for the loss of NKCC1. Moreover, the models suggest that the moderate reduction in ECS shrinkage is also partly due to the so-called transmembrane sodium cycle [47] where the influx of sodium is balanced by the sodium outflux through the Na/K/ATPase pump. This keeps [Na^+^]_i_ low even at activity-induced increase of Na^+^ influx (in fact [Na^+^]_i_ is lowered by high [K^+^]_o_, see Figure S4), preventing the intracellular osmolarity from reaching high levels which in turn limits water inflow. Blocking the transport of sodium through knockout of NKCC1 thus has only a limited effect on [Na^+^]_i_ (typically a reduction of 0.5–1 mM, see Figure S5), and, consequently, on volume shrinkage.

In our ECS-glia model system we assume that ECS shrinkage is accompanied by a corresponding astrocyte swelling, and that there is no water exchange with neurons. As the osmolarity of the ECS according to our calculations (Figure S4) does not change very much during shrinkage, and assuming that Na^+^ and K^+^ flow in and out of the neuron in approximately a 1∶1 ratio during excitation [9],[24], a significant import of water into the neuron does not seem likely. This is in accordance with the fact that most central nervous system neurons do not appear to have functionally operative water channels. Moreover, Ransom et al. [12] observed that repetitive activity in the optic nerve of rats caused shrinkage of the ECS due to cellular swelling only after astrocytes had proliferated and differentiated postnatally. Aitken et al. [48] reported that in one species of hippocampal neurons (CA1 pyramidal neurons) detectable volume changes did appear several minutes after the cells had been exposed to osmotic stress. Finally, by monitoring neuronal volume under osmotic stress in real time by 2-photon laser scanning microscopy (2PLSM), Andrew et al. [49] reported that pyramidal somata, dendrites, spines and cerebellar axon terminals maintained their volume while the grey matter swelled and shrinked as expected. If this applies to other neuronal types as well, and since astrocytes are capable of a substantial volume change in a matter of a few seconds [11],[13],[25],[47], it seems reasonable that the astrocyte is the main water sink during ECS shrinkage.

It should be emphasized that given the current state of knowledge, our results do not rule out other interpretations or contributions from other membrane processes. For instance, we have disregarded the possible role of the glutamate-, sodium- and potassium-carrying cotransporter EAAT1, known to be associated with active water transport [40] in grey matter, and the so-called chloride stretch channels [50] known to act as valves allowing chloride efflux from the swelling astrocyte, thus lowering [Cl^−^]_i_ and influencing intracellular osmolarity during swelling. Several studies suggest a role for the glial inwardly rectifying channel Kir4.1 in ECS potassium clearance [51]–[56]. Due to our observation that K^+^ never flows into the astrocyte through voltage-gated channels (e.g Kir or more conventional potassium channels), we did not include the Kir channel in our model. The model without cotransporters (mc1) can only provide ion concentrations consistent with empirical data when the ECS shrinkage level is inconsistent with empirical data. We addressed the question whether consistency can be more easily obtained if the potassium channel is replaced by Kir by repeating the numerical experiment for mc1 with the conductance of the potassium channel multiplied by a factor *f*_Kir_ [57] dependent on [K^+^]_o_ and the membrane potential. The result of this was a slight decrease in consistency rate and in mean shrinkage (Figure S6) showing that our model is not able to provide a role for Kir in potassium clearance. It can be that a model where the geometrical aspect is taken care of to a greater extent will be able to make a role for the Kir channels also in the potassium clearance phase. However, our present model provides several new predictions which we think need to be tested before one embarks on making more refined versions with more articulated geometrical representations.

All our model configurations are based on the assumption that ECS shrinkage is due to the redistribution of water and ions within the neuronal, astrocyte and ECS compartments. By this we disregard possible effects such as spatial potassium buffering [58] where K^+^ ions entering the ECS during neuronal activity are released from glia at some distant location where neurons are quiet. Gardner-Medwin [59] performed a theoretical analysis of the action of spatial buffering and reversible uptake in mammalian brain tissue during a ionophoretic point source release of potassium, and related the results to experimental cat neocortex data from Lux & Neher [60]. The author concluded that a reversible uptake is an essential component of total K^+^ clearance, slowing down the dynamics of [K^+^]_o_ and being especially relevant within short time spans after potassium release (order of magnitude 10 seconds). This suggests that even though spatial buffering may be an important process which needs to be dealt with, a model which emphasizes reversible uptake of potassium by astrocytes may serve as a good first approximation.

Our modelling effort identifies very clearly the need for measuring ECS shrinkage, membrane potential and ECS and astrocyte ion concentrations (potassium, sodium and chloride) in the wild type as well as NKCC1^−/−^ and AQP4^−/−^ individuals, in baseline and in excited conditions, and for obtaining data on neuronal water, sodium and chloride uptake during activation. Even though some of these measurements are experimentally challenging, most of them are within reach with current technology. These data will probably require revisions of some of our prevailing conceptions. However, considering our current state of knowledge, we think we have succeeded in building a well constrained mathematical model framework that is sufficiently detailed to guide future key experiments and pave the way for more comprehensive astroglia-neuron interaction models for normal as well as pathophysiological situations.

## Methods

### Model Construction

The structure of the mathematical modelling framework is based on the conceptual model outlined in Figure 1. The model describes the time rate of change of the numbers of sodium, potassium, and bicarbonate ions in the ECS and in the astrocyte and the time rate of change of the ECS and the astrocyte volume by ordinary differential equations, and the extra- and intracellular chloride concentrations and the astrocyte membrane potential by algebraic equations. The model structure is presented here. Text S1 provides derivations of model premises and equations.

### Model Equations

Using basic physical laws and principles, we developed an ordinary deterministic differential equation model where all variables and parameters have a specific biological/physical interpretation. The model equations and details concerning numerical simulations are presented below, where the extracellular and the intracellular (astrocytic) variables are indexed with o and i, respectively. Assuming space-charge neutrality in both compartments, intra- and extracellular electroneutrality require that
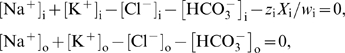
(1)where *X*_i_ is equal to the number of negatively charged impermeable ions trapped within the astrocyte divided by the astrocyte membrane area *A*, *z*_i_ is the average charge of these ions relative to the elementary charge, and *w*_i_ denotes the ratio of astrocyte volume to astrocyte area within the region of interest. Empirical data [9],[11] show that the ion species in eq. (1) are sufficient to achieve extracellular electroneutrality, hence the presence of impermeable ions in the ECS is neglected. The number (*N*) of each ion species *S* (Na^+^, K^+^, Cl^−^, HCO_3_^−^) per unit astrocyte area in the astrocyte compartment is given by the product
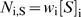
(2)and the expression for the extracellular compartment is structurally identical. The model equations for the time rate of change of the numbers of the respective intra- and extra-cellular ions read
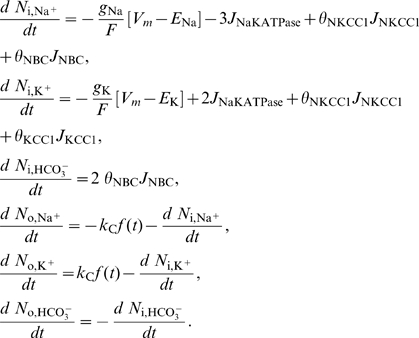
(3)


Here, extra- and intracellular chloride concentrations are determined by electroneutrality conditions (1), the Nernst potential of each of the four ion species is defined by
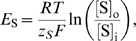
(4)
*V_m_* is the momentary membrane potential, *J*_NaKATPase_ is the sodium-potassium pump flux per membrane area depending on [Na^+^]_i_ and [K^+^]_o_ (see below), the constants *g*_Na_, *g*_Cl_, *g*_K_ are specific ion conductances for the respective species, *F* is Faraday's constant, *z_S_* is the valence of ion species *S*, *J*_NKCC1_, *J*_KCC1_, *J*_NBC_ are the electrochemically induced ion flux per membrane area through the NKCC1, KCC1, and NBC cotransporters, respectively (see below), and *k*_C_ *f(t)* denotes the time dependent incremental flux rate per membrane area of potassium and sodium between the ECS and the neuron per area (see Text S1). The symbols 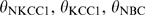 are given the value 0 or 1 according to which cotransporters are to be included in the model. Note that eqs.(3) imply that the total number of HCO_3_^−^ ions and the sum of the number of K^+^ and Na^+^ ions in the ECS and the astrocyte are conserved at any time. Ion fluxes through KCC1, NKCC1 [61] and NBC [45],[62] are modeled in a Nernst-like fashion, i.e.
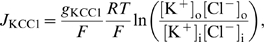
(5)

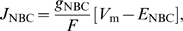
(6)

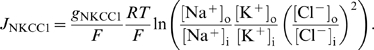
(7)


Here, *g*_NKCC1_, *g*_KCC1_ and *g*_NBC_ are the conductances per unit area for the NKCC1, the KCC1 and NBC cotransporter, respectively. The reversal potential of NBC is
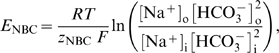
(8)where *z*_NBC_ is the effective valence of the NBC cotransporter complex, here taken to be −1, setting *z_NBC_* = −(*n*−1) = −1 where *n* is the stoichiometry, and adopting *n* = 2.

The Na/K/ATPase pump pumps 3 Na^+^ ions out of the astrocyte for each 2 K^+^ ions being pumped in. Simplifying the expression of Luo and Rudy [63], the Na/K/ATPase pump rate per area *J*_NaKATPase_ (given in mol s^−1^ cm^−2^) may be described as a function of the concentrations [Na^+^]_i_ and [K^+^]_o_, i.e.
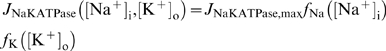
(9)where *f*_Na_ and *f*_K_ are [63]

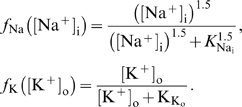
(10)The sodium and potassium fluxes per area through the astrocyte membrane are then 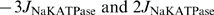, respectively (here we have chosen the inward direction to be positive), and the parameters *K*_Nai_ and *K*_Ko_ have dimension mM.

The assumed electroneutrality condition demands that the algebraic sum of all electric currents into the astrocyte has to be zero at every instant. The astrocytic membrane potential *V*_m_ is then given by solving the resulting equation with respect to *V*_m_;
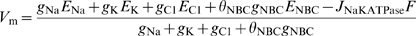
(11)


The rate of change of the astrocytic volume relative to its surface area, *w*_i_ = *v*_i_/*A*, is, by assumption, equal to the water flux (relative to surface area) through the osmosensitive channel AQP4 and the lipid membrane, i.e. the rate of change is proportional to the osmolarity gradient between the intra- and extracellular compartments;
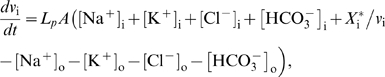
where *L_p_* is the total water permeability per unit area of the astrocyte membrane, and 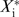 is the number of impermeable molecules in the astrocytes. If we introduce *w*_i_ for the ratio *v*_i_/*A*, assume constant surface area *A* [64] and set 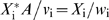, we obtain the area- and volume-independent formulation
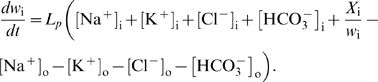
(12)


When water transport through NKCC1 is considered, an additional term *bJ*_NKCC1_ is included on the right hand side of eq. (12), where *b* is chosen such that the term describes a flux of 500 water molecules for every K^+^ and Na^+^ ion and for every two Cl^−^ ions, in accordance with numbers given by Zeuthen and MacAulay [65]. Finally, we assume that the total volume of ECS and astrocyte is constant, i.e.
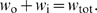
(13)


### Parameterization

Values for some of the baseline intracellular and extracellular ion concentrations, the baseline membrane potential (*V*_m_) and some of the parameters of the model were obtained from available experimental data. Taking into consideration that the empirically determined parameter values (including concentrations) come from various studies and different experimental settings, we chose a simulation approach where the value for each of the selected parameters was randomly drawn from a uniform distribution around the set point value.

Values for the baseline intracellular potassium and sodium concentrations 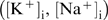 and extracellular potassium, sodium and bicarbonate concentrations 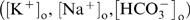 were drawn at random from the interval (0.9[S]_i,o_, 1.1[S]_i,o_), where [S]_i,o_ is the empirical baseline ion concentration of ion S (specified in Text S1). In accordance with available empirical data [14],[64] the baseline membrane potential (*V*_m_) was set equal to −85 mV. The initial astrocyte volume to area ratio was sampled at random from the interval (1/13 µm, 1/27 µm). The initial ratio of astrocyte to ECS volume, the Michaelis-Menten constants appearing in the expressions for the Na/K/ATPase pump rate (*K*_m,Nai_, *K*_m,Ko_), the sodium and chloride conductances (*g*_Na_, *g*_Cl_), the cotransporter conductances *g*_NKCC1_, *g*_KCC1_, *g*_NBC_ and the AQP4 permeability (*L_p_*) were all obtained from available experimental data (see Text S1) and sampled at random from (0.5*P*,1.5*P*), where *P* denotes the mean empirical value of any of the above parameters. Remaining parameters and ion concentrations were derived from the model equations to make certain that the model is in a steady state at baseline conditions (see Text S1). Initial osmotic equilibrium was ensured by adjusting the total charge of intracellular large anions not able to traverse the astrocyte membrane (*X*_i_).

Each simulation started with sampling a specific set of values from the given distributions and making estimations of all other parameters by use of the baseline steady-state equations (see Text S1) in order to calculate the excited steady state situation. For simulation of the time evolution, neural excitation was mimicked by an efflux of K^+^ to the ECS and a simultaneous equal influx into the neuron of Na^+^ from the ECS starting at *t* = 10 s and ending when an empirically relevant quantity of ions (taken as the integral over the given time interval of the flux *k_C_ f(t)*) had traversed the membrane. This quantity was estimated using available ion concentration measurements (see Text S1). To estimate the temporal profile *f(t)* of the K^+^ efflux/ Na^+^ influx rate we assumed that it was the shape of a beta distribution with parameters *a* = 2 and *b* such that the profile is optimized according to two criteria on the potassium concentration profile [K^+^]_o_(*t*); (i) the time from start until attaining maximum level should be 5 s, and (ii) the level at *t* = 30 s should be 60% of the maximum level.

The process of reinstating the baseline condition was started at *t* = 30 s by the onset of ion fluxes in the opposite directions of those described above, but using a simple rectangular temporal profile. In this way, we mimic phenomenologically the events following the termination of the period of enhanced neural activity. We let this period be equal to the interval of enhanced neuronal activity leading to the return to the baseline condition. Choosing a longer period would increase the time it takes to return to the baseline state but would not affect the shrinkage. However, the predicted degree of ECS shrinkage depends on the duration of the high-frequency firing period. Based on described experimental conditions [9],[11],[13], we started to reinstate the baseline condition after mimicking high-frequency firing for 20 s.

In order also to constrain the temporal model behaviour by observational data on the excited state we designed from the available literature a list of empirically valid ion concentration values during enhanced neural activity: (i) 6 mM<[Na^+^]_i_<12 mM [33]; (ii) 130 mM<[Na^+^]_o_<160 mM [9],[11],[13]; (iii) 130 mM<[Cl^−^]_o_<160 mM [9],[11],[13]; (iv) 6 mM<[K^+^]_o_<10 mM [9],[11]. We demanded at least two independent measurements of a given ion concentration before it could be used as a constraint. In some cases we also demanded that the relative ECS shrinkage during high neuronal activity should stay between 25 and 35% before a parameter set was judged to be empirically consistent.

### Model Translation

Although mathematical modelling has been identified as a valuable method for analysing large amounts of experimental data, unfortunately, inaccuracies often arise with the current method of mathematical model publication [66],[67]. Problems stem from the fact that models are developed and simulated in computer code, but require translation into text and equations for publication in journals. Replicating published results, or further developing a published model, is frequently impeded due to errors introduced during the publishing process such as typographical errors, missing parameters, or equations. Further, even when the model source code is made freely available, the code is often specific to a particular computer platform, or is incompatible with other modelling architectures.

CellML is an XML-based modelling language which provides an unambiguous method of defining models of biological processes [67]. It has been developed as a potential solution to the problems associated with publishing and implementing a mathematical model. The current model has been translated into CellML and the code is freely available for download from (http://www.cellml.org/models/ostby_oyehaug_einevoll_nagelhus_plahte_zeuthen_voipio_lloyd_ottersen_omholt_2008_version03). Model simulations can be run using the Physiome CellML Environment (PCEnv) or Cellular Open Resource (COR), two open source tools which can be downloaded from http://www.cellml.org/downloads/pcenv/ and http://cor.physiol.ox.ac.uk/, respectively.

## Supporting Information

Figure S1 Distribution of volume shrinkage and the potassium-volume shrinkage relation when different models include constitutive KCC1 and NKCC1. Left panels: Normalised histograms of the distributions of relative ECS volume shrinkage (in %) for the model configurations that are different than those depicted in Figure 2 in the main paper when KCC1 and NKCC1 are assumed to be constitutive (mc2, mc4 and mc5). The results were obtained by repeated numerical solution of steady state equations and only those parameter sets that satisfied the imposed ion concentrations constraints were used (see Methods in main text). Right panels: Corresponding scatter plots of excited state relative shrinkage and potassium concentrations using the same data as in the left panels. Since the upper limit of the shown relative shrinkage and the lower limit of the shown [K^+^]_o_ are set to 40% and 5 mM, respectively, some of the data are not displayed. Best linear fits are shown, and the corresponding slopes are 1.79, 2.42 and 3.68 in the H2, H4 and H5 cases, respectively (the H1 and H3 cases are the same as in Figure 2 in the main text). Moreover, for reasons of visualization only 10% of the data are depicted.
(0.20 MB EPS)

Figure S2 Distribution of volume shrinkage and the potassium-volume shrinkage relation for different models when we allow for chloride influx into the neuron. Left panels: Normalised histograms of the distributions of relative ECS volume shrinkage (in %) for the five model configurations (mc1–mc5). The results were obtained in the same manner as the results in Figure 2 in main paper, but, in addition, we allowed for a share θ (0<θ<0.5) of potassium neuronal efflux to be replaced by chloride neuronal influx. Right panels: Corresponding scatter plots of excited state relative shrinkage and potassium concentrations using the same data as in the left panels. Since the upper limit of the shown relative shrinkage and the lower limit of the shown [K^+^]_o_ are set to 40% and 5 mM, respectively, some of the data are not displayed. Best linear fits are shown, and the corresponding slopes are 2.76 (1.97), 2.43 (1.70), 4.47 (3.11), 3.43 (2.74) and 5.50 (4.08), respectively (values for the zero chloride flux case are given in parenthesis for comparison). For reasons of visualization only 10% of the data are depicted. Repeating the exercise adding a chloride neuronal influx of the same magnitude as above, but this time increasing the sodium influx accordingly to maintain electroneutrality, gave very similar results (data not shown).
(0.29 MB EPS)

Figure S3 Distribution of the share θ of potassium neuronal efflux that is replaced by chloride influx. Left panels: Normalised histograms of the distributions of the share θ of potassium neuronal efflux that is replaced by chloride for the five model configurations (mc1–mc5). The results were obtained by repeated numerical solution of steady state equations and only those parameter sets that satisfied the imposed ion concentrations constraints were used (see Methods). Right panels: Normalised histograms of the distributions of θ when those parameter sets that in addition to satisfying the imposed ion concentrations constraints satisfied the constraint that the relative volume shrinkage were in the range (25%, 35%).
(0.06 MB EPS)

Figure S4 Predicted ion concentration dynamics in wild type and gene knockouts. Predicted activity-dependent ion concentrations (A–G), membrane potential (H) and extracellular osmolarity (I) in wild type obtained by simulations on model H3 (blue) H4 (red) and H5 (black). Simulations with NKCC1 as well as AQP4 knockout give similar profiles (not shown).
(0.09 MB EPS)

Figure S5 Predicted wild type and NKCC1 knockout sodium concentration dynamics in H3 and H4. Predicted activity-dependent sodium concentrations [Na^+^]_i_ in wild type (blue) and NKCC1 knockout (red) obtained by simulations on model H3 (A) and H4 (B).
(0.02 MB EPS)

Figure S6 Distribution of volume shrinkage and the potassium-volume shrinkage relation for the model configuration mc1 with the regular potassium channel replaced by Kir. Left panel: Normalised histogram of the distribution of relative ECS volume shrinkage (in %) for the model configuration mc1 with the regular potassium channel replaced by an inwardly rectifying Kir channel. See main paper and legend to Figure 2 for an explanation of the procedure giving the present data. Right panel: Corresponding scatter plots of excited state relative shrinkage and potassium concentrations using the same data as in the left panels. Since the upper limit of the shown relative shrinkage and the lower limit of the shown [K^+^]_o_ are set to 40% and 5 mM, respectively, some of the data are not displayed. Best linear fit is shown with corresponding slope 1.66. For reasons of visualization only 10% of the data are depicted.
(0.04 MB EPS)

Text S1 Astrocytic mechanisms explaining neural-activity-induced shrinkage of extraneuronal space. A detailed description of the parameterization of the model and eight supporting tables are included.
(0.11 MB PDF)
