# Supplementary material for: Astrocytic Mechanisms Explaining Neural-Activity-Induced Shrinkage of Extraneuronal Space
Source: PLoS Comput Biol. 2009 Jan 23;5(1):e1000272. doi: 10.1371/journal.pcbi.1000272 (PMC2613522; doi:10.1371/journal.pcbi.1000272)
Supplement: Text S1 — Astrocytic mechanisms explaining neural-activity-induced shrinkage of extraneuronal space. A detailed description of the parameterization of the model and eight supporting tables are included. (0.11 MB PDF) [file pcbi.1000272.s007.pdf]

Supporting Text S1: Astrocytic mechanisms explaining neural-activity-induced shrinkage of extraneuronal space

Parameterization

Estimates of empirically supported parameters

Membrane area

Calculation of the specific conductivity g of the different ion species with the non-specific conductivities G empirically given makes it convenient to use a mean value for the membrane area of an astrocyte. By using a mean value for the membrane capacitance C_m = 37 pF [1-3] together with the specific capacitance 1.0 μF/cm^2 [4, 5], we arrive at a mean membrane area of 3.7×10^{-2} cm^2.

g_Cl = *chloride conductance associated with passive* Cl^- *channels*

Averages of passive astrocyte membrane properties reported [6, 7]include values of chloride current I_Cl (in pA) vs. potential V (in mV) relative to a holding potential of 0 mV. We use this to calculate the total chloride conductivity G_Cl (in Ω^{-1}) by setting G_Cl = $\Delta I_\mathrm{Cl} / \Delta V_\mathrm{m}$. We use the value cited above for the average membrane area A, and obtain

$$\text{(S1)}\; g_\mathrm{Cl} = \frac{G_\mathrm{Cl}}{A} = \frac{G_\mathrm{Cl}}{3.7\times10^{-2}\mathrm{cm}^2}.$$

Values of G_Cl and calculated values of g_Cl are given in Table S1.

g_Na = *sodium conductance associated with passive* Na^+ *channels*

Averages of passive membrane properties reported [1, 8] include values of astrocyte membrane capacitance C_m from 24.2 to 52.1 pF with an average value of 37 pF. Measured values of $G_\mathrm{Na}/C_\mathrm{m}$ (G_Na is defined as G_Cl above) and calculated values for g_{Na} are listed in Table S2.

g_{NBC} = *conductance of the NBC cotransporter*

Munsch and Deitmer [9] measured Na^+ and HCO_3^- dependent membrane currents in leech glial cells. From the curve they obtained from measured membrane current I_m as a function of membrane potential V_m, the conductance is estimated as

$$\text{(S2)}\; G_{\text{NBC}} = \frac{\Delta I_{\text{m}}}{\Delta V_{\text{m}}} \approx \frac{70\,\text{nA}}{25\,\text{mV}} = 2.8\times 10^{-6}\,\Omega^{-1}.$$

We use as before a membrane area of 3.7×10^{-2} cm^2. This yields the specific NBC conductance

$$\text{(S3)}\; g_{\text{NBC}} = \frac{G_{\text{NBC}}}{A} \approx \frac{2.8\times10^{-6}\,\Omega^{-1}}{3.7\times10^{-2}\,\text{cm}^2} = 7.6\times10^{-5}\,\Omega^{-1}\,\text{cm}^{-2}.$$

L_p = *specific water permeability through passive water channels*

The literature gives a lower and an upper estimate for L_p, defined through eq. (12) in the main text. In order to compare with literature estimates, we need first to translate given values of number flux permeability P_f (given in cm/s) into corresponding values of L_p (defined through volume flux). Since <u>number flux</u> j = number of molecules/(area · time) is related to P_f through the equation

$$\text{(S4)}\; j = P_f\, \Delta c,$$

where j is the number flux and Δc is the concentration gradient giving rise to j, while eq. (12) in the main paper states the following expression for <u>volume flux</u> J or volume/time measured in cm^3/s (membrane area A is inserted here to yield total flux through area A):

$$\text{(S5)}\; J = L_p\, A\, \Delta\Pi,$$

the total volume of water passing through the membrane per unit time measured in cm^3/s can be expressed by:

$$\text{(S6)}\; J = \frac{\text{total volume}}{\text{time}} = P_f\, A\, \Delta c \times \frac{\text{volume}}{\text{molecule}} = L_p\, A\, \Delta\Pi.$$

This means that with P_f given, we may calculate L_p by means of the relation

$$\text{(S7)}\; L_p = \frac{P_f\, \Delta c \dfrac{\text{volume}}{\text{molecule}}}{\Delta\Pi} = P_f \times \frac{\text{number of molecules}}{\text{mmol}} \times \frac{\text{volume}}{\text{molecule}},$$

since Δc is given as (number of molecules)/volume and $\Delta\Pi$ is given in units of mmol/volume. Hence, we have by eq. (S7)

$$\text{(S8)}\; L_p = P_f \times 6\times10^{20}\,\text{mmol}^{-1} \times 1.0\frac{1}{\text{kg}} \times 18 \times 1.66\times10^{-27}\,\text{kg} = \frac{P_f}{5.6\times10^4\,\text{mmol/l}}.$$

According to Gunnarson et al.[10], AQP4 channels in astrocytes have a P_f value of about 1.2×10^{-3} cm/s. By eq. (S8), this means a value of L_p of 2.1×10^{-8} (cm/s)(mM)$^{-1}$. A somewhat different estimate is given by Yang and Verkman [11]. Single channel water permeability for AQP4 is given as $(24\pm0.6)\times10^{-14}$ cm^3/s. With a density of AQP4 channels in mammalian astrocytes estimated as about 100 per μm^2 (unpublished), we arrive at the estimate

$$\text{(S9)}\; L_p = \frac{(24\times10^{-14}\,\text{cm}^3\,/\,\text{s})\times100\,\mu\text{m}^{-2}}{5.6\times10^4\,\text{mmol/l}} = 4.3\times10^{-8}\,\text{cm s}^{-1}\ \text{mM}^{-1}.$$

Potassium efflux and sodium influx

We seek expressions for the rate k_C of Na^+ and K^+ ions drawn into and expelled from the neuron, respectively, during transition from baseline to excited state. Both can be expressed in terms of the baseline ECS volume to astrocyte surface area ratio w_o^0 and intra- and extracellular ion concentrations.

We assume that the number of Na^+ ions taken up by and the number of K^+ ions that are expelled from the neuron are identical and equal to k_C (see below for a justification of this). This ensures that the total ECS and astrocyte charge is conserved. Further, we set the initial (baseline) astrocyte volume to ECS volume ratio w_i/w_o equal to p and assume that relative ECS volume shrinkage in the excited state is 30 %, implying that $w_o^1 \approx 0.7 w_o^0$ and $w_i^1 \approx (p+0.3)\,w_o^0$. Thus, in the baseline (BL) and excited (EX) states, respectively, we have

$$\text{(S10)}\; \begin{aligned} N_{K^+,\text{tot,BL}} &= [K^+]_o^0 w_o^0 + [K^+]_i^0 w_i^0 \approx w_o^0([K^+]_o^0 + p[K^+]_i^0), \\ N_{K^+,\text{tot,EX}} &= [K^+]_o^1 w_o^1 + [K^+]_i^1 w_i^1 \approx w_o^0(0.7[K^+]_o^1 + (p+0.3)[K^+]_i^1). \end{aligned}$$

Using $[K^+]_o^0$=3 mM, $[K^+]_i^0$=100 mM, $[K^+]_o^1$=7 mM, $[K^+]_i^1$=110 mM (where superscripts 0 and 1 refer to baseline and excited states, respectively), and subtracting the two we obtain

$$\text{(S11)}\; N_{K^+,\text{tot,EX}} - N_{K^+,\text{tot,BL}} \approx w_o^0 \times (1+0.29p)\times 34.9\ \text{mM},$$

which is the expelled K^+ per membrane area to the ECS from the neuron.

In addition to the quantity $w_o^0 \times (1+0.29p)\times 34.9$ mM, the flux rate per unit area k_C to be determined depends on the duration Δt of enhanced neural activity. The total amount of potassium (per unit area) expelled from the neuron during the time interval is equal to $k_C\Delta t$. Consequently, using $w_o^0 = w_i^0 / p$, $w_i^0 = 1/20\,\mu$m, Δt = 20 s and p = 2,

$$\text{(S12)}\ k_{\mathrm{C}} \approx \frac{w_{\mathrm{o}}^{0}(1+0.29p)\times 34.9\,\mathrm{mM}}{\Delta t} = \frac{w_{\mathrm{i}}^{0}}{\Delta t}\left(\frac{1}{p}+0.29\right)\times 34.9\,\mathrm{mM} \approx 0.069\,\mu\mathrm{m\ mM\ s^{-1}}.$$

In the model, we assume that the instantaneous potassium efflux (and sodium influx) is $k_C f(t)$ (or $-k_C f(t)$ in the sodium case), where the function $f(t)$ has unity integral and is found by requiring that $[K^+]_o$ has the temporal shape of a beta-distribution;

$$P(x) = \frac{(1-x)^{\beta-1}x^{\alpha-1}}{B(\alpha,\beta)},$$

where $B(\alpha,\beta)$ is the normalization constant that ensures $\int_0^1 P(x)dx = 1$. In our simulations we set α = 2 and estimate β according to some preset conditions (see Methods in main paper).

Justification of setting the Na^+ influx rate equal to K^+ efflux rate

By repeating the above for sodium, using $[\mathrm{Na}^+]_{\mathrm{o}}^{0}$=140 mM, $[\mathrm{Na}^+]_{\mathrm{i}}^{0}$=15 mM, $[\mathrm{Na}^+]_{\mathrm{o}}^{1}$=133 mM, $[\mathrm{Na}^+]_{\mathrm{i}}^{1}$=10 mM (where, again, superscripts 0 and 1 refer to baseline and excited states, respectively), the ratio K^+ efflux rate to Na^+ influx rate is

$$\frac{\mathrm{K^+\ efflux\ rate}}{\mathrm{Na^+\ influx\ rate}} \approx 0.795\frac{1+0.29p}{1+0.11p},$$

which lies in the range (0.92,1.10) for $1 \le p \le 3$, for p = 2 the ratio is 1.03 and the ratio is equal to 1 for p = 1.70. This demonstrates that the assumption that potassium and sodium fluxes are equal in magnitude is not contradicted by empirical data.

Other parameters

We also mention the flux parameters of NKCC1 and KCC1, g_{NKCC1} and g_{KCC1}, the astrocyte area to ECS volume v_i/A, the ECS to astrocyte volume v_o/v_i and the sodium-potassium pump parameters K_{Nai} and K_{Ko} (values of these parameters and associated references are listed in Table S8).

Estimation of initial ion concentrations

In order to solve the model equations some baseline ion concentrations were set (according to empirical data, see Table S3) and others were estimated. In the latter category, $[Cl^-]_o$ and $[Cl^-]_i$ were estimated using the ECS electroneutrality condition and the assumption that in the baseline state E_{Cl} is equal to the membrane potential, respectively (assuming that

KCC1 and/or NKCC1 are constitutive the latter estimation had to be altered). Further, $[HCO_3^-]_i$ was estimated assuming that E_{NBC} is equal to the baseline membrane potential.

Number of intracellular impermeable molecules X_i

The number per unit area of impermeable molecules X_i (within the astrocyte) was estimated using osmotic equilibrium at baseline steady state;

$$X_i = w_i^0([Na^+]_o - [Na^+]_i + [K^+]_o - [K^+]_i + [Cl^-]_o - [Cl^-]_i + [HCO3^-]_o - [HCO3^-]_i).$$

This estimate would have been different if X_i had been calculated based on intracellular electroneutrality. This apparent contradiction is resolved by presuming that intracellular impermeable molecules have an average charge z_i that is estimated using the first of eq. (1) in the main paper under baseline conditions. The quantity z_i does not affect the behaviour of the model equations, since the membrane potential at any instant is found by using the electroneutrality condition

$$\text{(S13)} \quad \frac{dN_{i,Na^+}}{dt} + \frac{dN_{i,K^+}}{dt} = \frac{dN_{i,Cl^-}}{dt} + \frac{dN_{i,HCO_3^-}}{dt}$$

and substitute the expressions from eqs. (3) in the main paper.

Potassium conductance and $J_{NaKATPase,max}$

The values of the maximum Na/K/ATPase pump rate $J_{NaKATPase,max}$ and g_K were estimated as solutions of the baseline steady state equations (obtained by setting the derivatives in Eqs. (3) in the main paper equal to zero and using baseline values of ion concentrations and membrane potential),

$$\text{(S14)} \quad \begin{aligned} &-\frac{g_{Na}}{F}[V_m - E_{Na}] - 3J_{NaKATPase} = 0, \\ &-\frac{g_K}{F}[V_m - E_K] + 2J_{NaKATPase} = 0. \end{aligned}$$

Note that the rate $J_{NaKATPase,max}$ is obtained directly from the first of eqs. (S14), and that g_K then follows directly from the second of eqs. (S14). The values for the parameters not mentioned here were set (according to the values given in Table S8).

Supporting tables

Table S1 Measured values of G_{Cl} and corresponding calculated values of g_{Cl}.

Reference	G_{Cl} (Ω^{-1})	g_{Cl} ($\Omega^{-1}cm^{-2}$)
Makara et al., 2003 [7]	2.0×10^{-9}	5.4×10^{-5}
Ferroni et al., 1997 [6]	2.7×10^{-8}	7.3×10^{-4}

Table S2 Measured values of C_m, G_{Na} / C_m and corresponding calculated values of g_{Na}.

Reference	Species	C_m (pF)	G_{Na} / C_m (pS/pF)	g_{Na} ($\Omega^{-1}cm^{-2}$)
Bordey and Sontheimer, 2000 [2]	Rats, hippocampus dentate gyrus	24.2	462	4.9×10^{-4}
Bordey and Sontheimer, 2000 [2]	Rats, cortex	34.3	301.3	3.0×10^{-4}
Bordey et al., 2000 [3]	Rats, cortical layer I	41.4	99.6	1.0×10^{-4}
Bordey and Sontheimer, 1998 [1]	Human, hippocampus/cortex	52.1	32.3	3.2×10^{-5}
Bordey and Sontheimer, 1998 [1]	Rats, hippocampus	32.0	88.7	8.9×10^{-5}

Table S3 Baseline ion concentrations with references.

	Concentration (mM)	Reference(s)
$[K^+]_o$	3	Dietzel et al.1982[12]; Walz and Hertz 1983[13]; Dietzel and Heinemann 1986[14]; Lux et al. 1986[15]; Rose and Ransom 1996[16]
$[Na^+]_o$	146	Dietzel and Heinemann 1986[14]; Dietzel et al.1982[12]
$[HCO_3^-]_o$	15	Tong and Chesler 2000[17]; Boussouf et al. 1997[18]; Sohma et al. 2000[19]
$[K^+]_i$	100	Walz and Hertz 1983[13]; Stys et al. 1997[20]
$[Na^+]_i$	15	Rose and Ransom 1996[16]

Table S4 Consistency rates and relative volume shrinkage. Consistency rates (CR) and relative volume shrinkage (RS) in the excited state given by model configurations mc2, mc4 and mc5 under the two different constraints situations (ion concentrations only (EC1) and ion concentrations plus ECS shrinkage in the 25-35 % range (EC2)). Here, data are obtained assuming that KCC1 and NKCC1 are constitutive.

	EC1		EC2	
	CR_1 (%)	RS_1 (%)	CR_2 (%)	RS_2 (%)
H2	5.2	10.8±4.5	0.008	25.3±0.2
H4	15.1	13.1±4.7	0.16	26.6±1.4
H5	36.4	18.7±6.6	6.1	28.6±2.6

Table S5 Ion concentrations in the excited state. Ion concentrations in the excited state (given as mean ± sd, mean ± sd of baseline ion concentrations are provided in parentheses for comparison) for the parameter sets that satisfy the ion concentration constraints plus ECS shrinkage in the 25-35 % range in the model configurations mc3 - mc5.

	H3	H4	H5
$[K^+]_o$ (mM)	9.4 ± 0.5 (2.9 ± 0.1)	9.2 ± 0.6 (2.9 ± 0.1)	8.6 ± 0.9 (3.0± 0.2)
$[K^+]_i$ (mM)	110 ± 5 (100 ± 6)	110 ± 5 (100 ± 6)	110 ± 5 (100 ± 6)
$[Na^+]_o$ (mM)	141 ± 7 (148 ± 7)	148 ± 4 (154 ± 4)	143 ± 7 (149 ± 7)
$[Na^+]_i$ (mM)	8.5 ± 1.0 (14.9 ± 0.9)	8.8 ± 1.0 (14.9 ± 0.9)	9.0 ± 1.0 (15.0 ± 0.9)
$[Cl^-]_o$ (mM)	142 ± 7 (135 ± 7)	137 ± 4 (142 ± 4)	142 ± 7 (136 ± 7)
$[Cl^-]_i$ (mM)	15.2 ± 1.1 (5.1 ± 0.3)	19 ± 1 (5.3 ± 0.1)	17 ± 2 (5.1 ± 0.3)
$[HCO_3^-]_o$ (mM)	8.5 ± 0.6 (15.1 ± 0.9)	20 ± 1 (14.9 ± 0.8)	9.1 ± 0.8 (15.0 ± 0.9)
$[HCO_3^-]_i$ (mM)	11.4 ± 0.8 (9.2 ± 0.6)	8.4 ± 0.5 (9.2 ± 0.6)	11.3 ± 0.8 (9.1 ± 0.6)

Table S6 Consistency rates and relative volume shrinkage when there is a neuronal influx of chloride. Consistency rates (CR) and relative volume shrinkage (RS) in the excited state given by model configurations mc1 to mc5 under the two different constraints situations (ion concentrations only (EC1) and ion concentrations plus ECS shrinkage in the 25-35 % range (EC2)) for the case where a share of the potassium neuronal efflux is replaced by a chloride influx. The case where the potassium efflux is unaltered and a flux of the same magnitude is added to the sodium influx gives very similar results (data not shown).

	EC1		EC2	
	CR_1 (%)	RS_1 (%)	CR_2 (%)	RS_2 (%)
H1	10.9	14.8±5.9	0.642	27.9±2.3
H2	5.9	14.2±5.7	0.278	27.8±2.1
H3	26.2	22.1±9.4	5.8	29.2±2.8
H4	11.6	17.9±6.4	1.6	28.5±2.6
H5	25.5	27.0±11.0	7.3	29.5±2.9

Table S7 Empirically supported concentration data in baseline and excited states. Summary of empirically supported concentration data in baseline and excited states (given as “concentration in baseline/concentration in excited state”) suitable for comparison with model outputs.

Reference	$[K^+]_o$ (mM)	$[Na^+]_o$ (mM)	$[Cl]_o$ (mM)	$[K^+]_i$ (mM)	$[Na^+]_i$ (mM)	$[Cl^-]_i$ (mM)
Dietzel et al., 1982 a) [12]	3/10	146/139	149/156	-/-	-/-	-/-
Walz and Hertz, 1983 b) [13]	3/12	-/-	-/-	80/118	28/22	23/28
Dietzel and Heinemann, 1986 c) [14]	3/7	146/142	148/152	-/-	-/-	-/-
Lux et al., 1986 d) [15]	3/9	149/142	144/150	-/-	-/-	-/-
Rose and Ransom, 1996 e) [16]	3/4, 8	-/-	-/-	-/-	14.6/12.1, 9.5	-/-
Bekar and Walz, 2002 f) [21]	-/-	-/-	-/-	-/-	-/-	29/-
Stys et al., 1997 g) [20]	-/-	-/-	-/-	122/-	39/-	25/-

a) In vivo stimulation through Ag/AgCl balls on cortical surface or by electrical stimuli, recording micro-electrodes in the sensi-motor cortex of cats. b) Radiotracers in primary cultures of astrocytes, result of increase of $[K^+]_o$. c) In vivo measurements on cat brain, ex vivo measurements on rat hippocampal slices, physiological, electrical and chemical stimuli. d) In vivo experiments on cats, cortex, thalamus, spinal cord, dorsal-column nuclei, hippo-campus, electrical stimulation. e) In vitro measurements on astrocytes in standard saline, emission fluorescence measurements of intracellular Na^+. f) Cell culture of hippocampal astrocytes from rats in perfusion chamber, measurement of effect of $GABA_A$ agonist muscimol on K^+ current. g) Interface brain slice chamber with dissected optic nerves from rats, electron probe X-ray measurements of water content and concentrations.

Table S8 Parameters of the model with values. Parameters of type “empirical” have been retrieved directly from the literature, while the “estimated” parameters have been calculated using data found in the literature.

Parameter	Value	Type	Explanation and reference
R	8.315 J mol^{-1} K^{-1}	Physical constant	Universal gas constant
F	9.649×10^{4} C mol^{-1}	Physical constant	Faraday’s constant
T	300 K	Physical constant	Temperature
e	1.6×10^{-19} C	Physical constant	Elementary charge
g_{Na}	1×10^{-4} Ω^{-1} cm^{-2}	Empirical/estimated	Sodium conductivity per membrane area
g_{Cl}	1×10^{-4} Ω^{-1} cm^{-2}	Empirical/estimated	Chloride conductivity per membrane area
$w_{\mathrm{i}}=v_i/A$ [a)]	1/20 µm	Empirical	Baseline ratio of astrocyte volume to area [22]
v_o/v_{i} [b)]	0.5	Empirical	Baseline ratio of ECS volume to astrocyte volume [23]
L_p	2×10^{-8} cm s^{-1} (mM)$^{-1}$.	Empirical/estimated	Permeability of the astrocyte membrane
g_{NKCC1}	2×10^{-6} Ω^{-1} cm^{-2}	Empirical	Flux parameter of NKCC1 cotransporter [24]
g_{KCC1}	7×10^{-5} Ω^{-1} cm^{-2}	Empirical	Flux parameter of KCC1 cotransporter [24]
g_{NBC}	8×10^{-5} Ω^{-1} cm^{-2}	Empirical/estimated	Flux parameter of NBC cotransporter (Ω^{-1} cm^{-2})
k_{C}	≈0.069 µm mM s^{-1} (see earlier section)	Empirical/estimated	Quantity describing the rate of neuronal efflux of K^+ and influx of Na^+
K_{Nai}	10 mM	Empirical	Threshold value for $[Na+]_i$ in Na/K/ATPase model [25]
K_{Ko}	1.5 mM	Empirical	Threshold value for $[K+]_o$ in Na/K/ATPase model [25]

a) Data from rat dentate gyrus (18.9 - 33.0 μm^{-1}, mean 26.2 μm^{-1} [22]), Bergmann glia (cerebellum) (13 – 15 μm^{-1} [26]) and rats, gliotic cortex, reactive astrocytes (5.37 ± 0.62 μm^{-1} [27]). b) Estimates: 0.44 based on ECS volume fraction = 20% and glial volume fraction = 45 % [23, 28], 0.25 based on $V_i = 3.8 \times 10^{-8}$ cm^3, $V_o = 9.50 \times 10^{-9}$ cm^3 [5]and 0.67 [24].

References

1. Bordey A, Sontheimer H. (1998) Properties of human glial cells asociated with epileptic seizure foci. Epilepsy Res 32: 286-303.

2. Bordey A, Sontheimer H. (2000) Ion channel expression by astrocytes in situ: comparison of different CNS regions. Glia 30: 27-38.

3. Bordey A, Lyons SA, Hablitz JJ, Sontheimer H. (2000) Electrophysiological characteristics of reactive astrocytes in experimental cortical dysplasia. J Neurophysiol 85(4): 1719-1731.

4. Nelson P. (2004) Biological Physics. New York: Freeman & Company.

5. Yi CS, Fogelson AL, Keener JP, Peskin CS. (2003) A mathematical study of volume shifts and ionic concentration changes during ischemia and hypoxia. J.Theor.Biol. 220(1): 83-106.

6. Ferroni S, Marchini C, Nobile M, Rapisarda C. (1997) Characterization of an inwardly rectifying chloride conductance expressed by cultured rat cortical astrocytes. Glia 21: 217-227.

7. Makara JK, Rappert A, Matthias K, Steinhäuser C, et al. (2003) Astrocytes from mouse brain slices express C1C-2-mediated Cl- currents regulated during development and after injury. Mol Cell Neurosci 23: 521-530.

8. Bordey A, Sontheimer H. (2000) Ion channel expression by astrocytes in situ: comparison of different CNS regions. Glia 30(1): 27-38.

9. Munsch T, Deitmer JW. (1994) Sodium-bicarbonate cotransport current in identified leech glial cells. J Physiol 474(1): 43-53.

10. Gunnarson E, Axehult G, Baturina G, Zelenin S, et al. (2005) Lead induces increased water permeability in astrocytes expressing aquaporin 4. Neuroscience 136: 105-114.

11. Yang B,Verkman AS. (1997) Water and glycerol permeabilities of aquaporins 1-5 and MIP determined quantitatively by expression of epitope-tagged constructs in Xenopus oocytes. J Biol Chem 272(26): 16140-16146.

12. Dietzel I, Heinemann U, Hofmeier G, Lux HD. (1982) Stimulus-induced changes in extracellular Na+ and Cl- concentration in relation to changes in the size of the extracellular space. Exp Brain Res 46(1): 73-84.

13. Walz W, Hertz L. (1983) Intracellular ion changes of astrocytes in response to extracellular potassium. J Neurosci Res 10(4): 411-423.

14. Dietzel I,Heinemann U. (1986) Dynamic variations of the brain cell microenvironment in relation to neuronal hyperactivity. Ann NY Acad Sci 481: 72-86.

15. Lux HD, Heinemann U, Dietzel I. (1986) Ionic changes and alterations in the size of the extracellular space during epileptic activity. Adv Neurol 44: 619-639.

16. Rose CR, Ransom BR. (1996) Intracellular sodium homeostasis in rat hippocampal astrocytes. J Physiol (Lond) 491(2): 291-305.

17. Tong CK, Chesler M. (2000) Modulation of spreading depression by changes in extracellular pH. J Neurophysiol 84(5): 2449-2457.

18. Boussouf A, Lambert RC. (1997) Voltage-dependent Na+-HCO3- cotransporter and Na+/H+ exchanger are involved in intracelular pH regulation of cultured mature rat cerebellar oligodendrocytes. Glia 19: 74-84.

19. Sohma Y, Gray MA, Imai Y, Argent BE. (2000) HCO3- transport in a mathematical model of the pancreatic ductal epithelium. J Membrane Biol 176: 77-100.

20. Stys PK, Lehning E, Saubermann AJ, LoPachin Jr. RM. (1997) Intracellular concentrations of major ions in rat myelinated axons and glia: Calculations based on electron probe X-ray microanalyses. J Neurochem 68(5): 1920-1928.

21. Bekar LK,Walz W. (2002) Intracellular chloride modulates A-type potassium currents in astrocytes. Glia 39: 207-216.

22. Hama K, Arii T. (2004) Tri-dimensional morphometric analysis of astrocytic processes with high voltage electron microscopy of thick Golgi preparations. J Neurocyt 33: 277-285.

23. Anderson AW, Xie J, Pizzonia J, Bronen RA, et al. (2000) Effects of cell volume fraction changes on apparent diffusion in human cells. Magn Reson Imaging 18: 689-695.

24. Dronne MA, Boissel JP, Grenier E. (2006) A mathematical model of ion movements in grey matter during a stroke. J Theor Biol 240(4): 599-615.

25. Luo CH, Rudy Y. (1994) A dynamic model of the cardiac ventricular action potential. I. Simulations of ionic currents and concentration changes. Circ Res 74(6): 1071-1096.

26. Grosche J, Matyash V, Möller T, Verkhratsky A, et al. (1999) Microdomains for neuron-glia interaction: parallel fiber signaling to Bergmann glial cells. Nat Neurosci 2(2): 139-143.

27. Anderova M, Antonova T, Petrik D, Neprasova H, et al. (2004) Voltage-dependent potassium currents in hypertrophied rat astrocytes after a cortical stab wound. Glia 48(4): 311-326.

28. Syková E, Vargová L, Prokopová S, Simonová Z. (1999) Glial swelling and astrogliosis produce diffusion barriers in the rat spinal cord. Glia 25: 56-70.
